# Plasma Lipids Associated with Endothelial Dysfunction in Patients with Type 2 Diabetes: A Cross-Sectional Study Using Lipidomic Analysis

**DOI:** 10.3390/ijms27198437

**Published:** 2026-09-22

**Authors:** Naohiro Taya, Naoto Katakami, Kazuo Omori, Shigero Hosoe, Mitsuyoshi Takahara, Kazuyuki Miyashita, Shugo Sasaki, Yutaka Konya, Sachiko Obara, Ayako Hidaka, Motonao Nakao, Masatomo Takahashi, Yoshihiro Izumi, Takeshi Bamba, Iichiro Shimomura

**Affiliations:** 1 Department of Metabolic Medicine, Graduate School of Medicine, The University of Osaka, 2-2, Yamadaoka, Suita 565-0871, Osaka, Japan; taya@endmet.med.osaka-u.ac.jp (N.T.); omori@endmet.med.osaka-u.ac.jp (K.O.); shosoe@endmet.med.osaka-u.ac.jp (S.H.); takahara@endmet.med.osaka-u.ac.jp (M.T.); kmiyas@endmet.med.osaka-u.ac.jp (K.M.); shugosasaki@endmet.med.osaka-u.ac.jp (S.S.); shimomura@endmet.med.osaka-u.ac.jp (I.S.); 2 Department of Diabetes Care Medicine, Graduate School of Medicine, The University of Osaka, 2-2, Yamadaoka, Suita 565-0871, Osaka, Japan; 3 Department of Laboratory Medicine, Graduate School of Medicine, The University of Osaka, 2-2, Yamadaoka, Suita 565-0871, Osaka, Japan; 4 Division of Metabolomics, Medical Research Center for High Depth Omics, Medical Institute of Bioregulation, Kyushu University, 3-1-1 Maidashi, Higashi-ku, Fukuoka 812-8582, Fukuoka, Japan; y_konya00@shimadzu-techno.co.jp (Y.K.); comonstock@hotmail.co.jp (S.O.); a.hidaka.uq@adm.fukuoka-u.ac.jp (A.H.); nakao@be-force.co.jp (M.N.); takahashi.masatomo.sci@osaka-u.ac.jp (M.T.); izumi.yoshihiro.sci@osaka-u.ac.jp (Y.I.); bamba@sysmet.med.osaka-u.ac.jp (T.B.); 5 Mass Spectrometry Center, Graduate School of Science, The University of Osaka, 1-1 Machikaneyama, Toyonaka 560-0043, Osaka, Japan; 6 Laboratory of Systems Metabolomics, Graduate School of Medicine, The University of Osaka, 2-2, Yamadaoka, Suita 565-0871, Osaka, Japan

**Keywords:** diabetic complication, endothelial dysfunction, flow-mediated vasodilation, lipidomics, phospholipids, type 2 diabetes

## Abstract

Patients with diabetes often develop abnormalities in lipid metabolism that can promote diabetic complications. Lipidomic studies have demonstrated that certain plasma lipid species are associated with the development of cardiovascular diseases. However, the plasma lipids associated with endothelial dysfunction remain unclear. To identify the lipids associated with flow-mediated vasodilation (FMD), an indicator of endothelial function, in patients with diabetes, we performed a cross-sectional analysis as a subanalysis of a previous lipidomic study. In total, 200 patients with type 2 diabetes were included in the analysis. A total of 55.0% were men, and the mean age (±standard deviation) was 61.1 ± 11.3 years. Their HbA1c and FMD values were 9.04 ± 1.79% (75.3 ± 19.5 mmol/mol) and 5.77 ± 2.55%, respectively. Supercritical fluid chromatography/mass spectrometry-based semi-targeted lipidomic analysis identified 349 lipids belonging to 16 lipid classes. We selected 13 distinctive lipids potentially associated with FMD, according to a sparse-group least absolute shrinkage and selection operator regularized regression model. Of these, seven phospholipids, including three phosphatidylethanolamine (PE) plasmalogens and three phosphatidylinositols (PIs), showed a positive association with FMD after adjusting for atherosclerotic risk factors. These findings may contribute to improving the assessment and management of endothelial dysfunction in patients with diabetes.

## 1. Introduction

Diabetes mellitus has a high global prevalence (10.5%), and the number of patients with diabetes is estimated to increase from 540 million to 780 million by 2045 [1]. This common chronic disease is a major risk factor for cardiovascular diseases (CVDs), such as myocardial infarction, peripheral artery disease, and stroke [2,3]. These CVDs are, in turn, some of the main causes of death in patients with diabetes, impose a substantial economic burden on them, and reduce their quality of life. Therefore, CVD is considered one of the crucial complications of diabetes.

CVD is often a clinical manifestation that develops as a result of the progression of atherosclerosis. Atherosclerosis begins at a subclinical stage, and various environmental and genetic factors promote its progression, eventually leading to CVD. Diabetes is one of the major factors that promote atherosclerosis, and accelerates its progression through multiple mechanisms, including endothelial dysfunction, inflammation, oxidative stress, and endothelial-to-mesenchymal transition [4,5]. Endothelial dysfunction plays an important role in the development of diabetes-accelerated atherosclerosis by promoting leukocyte and platelet adhesion, thrombosis, and inflammation [4,5,6,7]. It can be assessed in clinical settings using flow-mediated vasodilation (FMD), which is a well-established index of atherosclerosis and a predictor of CVDs [8,9]. Furthermore, non-invasive methods for assessing endothelial function, including FMD, have been widely used in research [10].

Patients with diabetes often develop abnormalities in lipid metabolism in addition to hyperglycemia, and these abnormalities can accelerate the progression of atherosclerosis. Indeed, lipid metabolic abnormalities commonly observed in patients with diabetes, such as increased levels of small dense LDL and postprandial hypertriglyceridemia, are associated with endothelial dysfunction [11,12]. Moreover, lipidomics has increasingly been applied in clinical research for a better understanding of disease pathophysiology and the detection of novel biomarkers [13], and lipidomic studies have revealed that various blood lipid species can promote atherosclerosis and predict CVDs [14,15,16]. For example, certain ceramides, sphingolipids and lysophospholipids are associated with CVD development [14,15,16,17,18,19]. Basic research has elucidated the mechanism by which ceramide promotes atherosclerosis, including impairing endothelial function by inhibiting nitric oxide production [20], and a recent observational study has revealed the association between plasma ceramide level and endothelial dysfunction [21]. Similarly, the other lipids associated with endothelial dysfunction can promote atherosclerosis by impairing endothelial function, and identifying these lipids can improve our understanding of the pathophysiology of atherosclerosis. Furthermore, the identified lipids might contribute to the assessment and treatment of endothelial dysfunction as potential biomarkers and novel therapeutic targets, respectively. However, the blood lipid species associated with endothelial dysfunction and FMD have not been fully elucidated.

To elucidate the mechanism underlying the development of diabetes-accelerated atherosclerosis and improve the prevention or treatment of it, we aimed to identify lipids associated with endothelial dysfunction in patients with diabetes.

## 2. Results

### 2.1. Patient Characteristics

This cross-sectional study used baseline data from a previous prospective observational study of patients with type 2 diabetes (T2D) [22]. Among the 240 participants in the original study, 40 were excluded because FMD was not assessed (Figure 1). The participants in this analysis had a lower prevalence of males, coronary artery disease (CAD), and diabetic neuropathy and a lower level of uric acid than those excluded (Table S1).

The clinical characteristics of the 200 patients included in this analysis are summarized in Table 1. In total, 55.0% were men, and the mean age (±standard deviation) was 61.1 ± 11.3 years. The duration of diabetes was 12.6 ± 9.4 years, and 65.0% had hypertension. They had a relatively high BMI of 27.53 ± 5.75 kg/m^2^ and poor glycemic control with a HbA1c level of 9.04 ± 1.79% (75.3 ± 19.5 mmol/mol). The LDL cholesterol level was 2.918 ± 0.910 mmol/L, and 43.0% of the participants were statin users. The FMD value was 5.77 ± 2.55%, and 55 patients (27.5%) were classified as having endothelial dysfunction based on the proposed criteria defining FMD < 4.0% as endothelial dysfunction [23]. Patients with endothelial dysfunction were older and had a higher urine albumin-to-creatinine ratio and a higher prevalence of current smoking, hypertension, and diabetic neuropathy than those without it.

### 2.2. Identification of the Lipid Associated with FMD

Plasma lipidomic analysis using supercritical fluid chromatography coupled with tandem mass spectrometry (SFC/MS/MS) identified 349 lipids belonging to 16 lipid classes (Table S2). From these, the distinctive lipids related to FMD were selected using a sparse-group least absolute shrinkage and selection operator (LASSO) regularized regression model. Consequently, 13 lipids belonging to four lipid classes, hexosylceramide (HexCer), phosphatidylcholine (PC), phosphatidylethanolamine (PE) and phosphatidylinositol (PI), were identified as the lipids potentially associated with FMD (Figure S1). Figure 2 shows the results of principal component analyses (PCAs) using the data of plasma lipid species. No difference in the principal components derived from all the lipids was observed among patients with and without endothelial dysfunction (Figure 2A). In contrast, although substantial overlap in those from the lipids selected by the sparse-group LASSO regularized regression analysis was observed, there was a slight difference in the first principal components (Figure 2B). These results suggested that relatively distinctive lipids related to FMD were selected.

Figure 3 shows the associations between the selected lipids and FMD evaluated using multivariable regression models. Seven lipids, including three PE plasmalogens (PE 16:0p_20:1, PE 16:0p_22:4, and PE 16:0p_22:5) and three PIs (PI 18:1_20:4, PI 19:0_20:4, and PI 20:0_20:4), showed a statistically significant positive association with FMD after adjusting for age, sex, duration of diabetes, smoking status, BMI, hypertension, HbA1c level, and statin use, whereas none showed a negative association. Similar results were observed in the sensitivity analysis restricted to patients without a history of CAD (Figure S2).

## 3. Discussion

The present study explored lipids associated with endothelial dysfunction in patients with T2D using semi-targeted plasma lipidomic analysis. We selected 13 distinctive lipids related to FMD according to the sparse-group LASSO regularized regression model and found that seven of these lipids were associated with FMD independent of atherosclerotic risk factors.

Atherosclerosis is associated with mortality, high economic burden, and reduced quality of life in patients with diabetes. As early diagnosis and prevention of atherosclerosis can improve the outcome of patients with T2D, the detailed mechanism underlying the development of diabetes-accelerated atherosclerosis should be further elucidated. The endothelium functions not merely as a structural barrier between the vascular lumen and vessel wall but also plays a crucial role in vascular homeostasis by regulating vasoreactivity, angiogenesis, immunomodulation, nutrient uptake and coagulation [6]. Endothelial dysfunction contributes to atherosclerosis in patients with diabetes [6,7], and its importance in the pathogenesis of atherosclerosis has been supported [24]. Although lipidomic studies have identified lipids related to cardiovascular outcomes or indices of atherosclerosis [14,15,16,17,18,19], the associations between blood lipid species and indices of endothelial dysfunction are not well understood. Therefore, the findings of the present study could provide important insights into the pathophysiology of endothelial dysfunction and atherosclerosis. In addition, they might contribute to identifying potential biomarkers of endothelial dysfunction and novel therapeutic targets for it.

Notably, all lipids that were identified as being associated with FMD in the present study were phospholipids, and their associations with FMD were positive. This suggests that some phospholipids may be associated with the prevention of diabetes-accelerated atherosclerosis by inhibiting endothelial dysfunction. Phospholipids comprise a surface monolayer of lipoproteins and regulate the binding of apolipoproteins and enzymes to lipoprotein surfaces [25,26], thereby regulating lipoprotein metabolism [27]. This phospholipid-induced change in lipoprotein metabolism can affect the development of atherosclerosis.

The lipids identified included three PE plasmalogens. Plasmalogens are glycerophospholipids that contain a vinyl–ether bond at the sn-1 position. Vinyl–ether bonds are susceptible to oxidation, suggesting that plasmalogens are antioxidant lipids. Certainly, plasmalogens were shown to inhibit the oxidation of cholesterol and LDL [28,29]. This antioxidant property of plasmalogens appears to contribute to protection against the development of atherosclerosis, which is supported by clinical studies. In a cross-sectional study of patients suspected of CAD, patients with coronary stenosis tended to have lower levels of serum plasmalogens than those without [30]. Another cross-sectional study revealed that the total plasma plasmalogen levels were lower in patients with unstable CAD than in those with stable CAD, whereas no significant difference was observed between the stable CAD group and healthy controls in this study [31]. Moreover, a cohort study showed that three plasmalogens (PC 16:0p_20:4, PE 16:0p_20:4, and PE 18:0p_20:4) in the plasma were associated with a decreased risk of future atherosclerotic CVD [32].

Similarly, the lipids identified in the present study included three PIs, and associations between decreased levels of plasma PIs and CVD were observed in previous lipidomic studies [32,33]. A cross-sectional study revealed that PI levels were consistently lower in patients with stable CAD than in healthy controls [33]. A longitudinal association between one PI (PI 16:0_18:2) and decreased risk of atherosclerotic CVD was also observed in the abovementioned cohort study [32]. PI plays a well-established role in cellular signaling related to atherosclerosis as a substrate of phosphoinositide 3-kinase, the signaling pathways of which are involved in inflammation, oxidative stress, endothelial dysfunction, proliferation of smooth muscle cells, and foam cell formation [34,35]. However, the mechanistic role of plasma PIs in atherosclerosis development remains unclear.

From the above, our findings are consistent with those of previous clinical studies [30,31,32,33]. Although these studies suggest an inhibitory role of plasmalogens and PIs in incident CVD, our findings additionally indicate that these lipid classes are associated with the prevention of atherosclerosis by inhibiting endothelial dysfunction. Furthermore, these preventive effects can be exerted from a relatively early stage, as our results remained consistent even when the analysis was restricted to patients without a history of CAD.

Interestingly, the fatty acid (FA) composition of the identified lipids is characterized by an enrichment of specific FAs: all the identified PE plasmalogens have FA 16:0 as FA constituents and all the PIs have FA 20:4. FA 16:0 (palmitic acid) is associated with increased risk of CVD, as supported by both epidemiological and basic research [36]. FA 20:4 (arachidonic acid) is known as a precursor of pro-inflammatory mediators, while its derivatives also include anti-atherosclerotic mediators [37,38], and associations between circulating and adipose tissue levels of arachidonic acid and cardiovascular outcomes were not observed in meta-analysis of observational studies [39]. These findings partly appear to contradict our findings, but the reason for this discrepancy remains unclear. Note that our identified lipids which have FA 16:0 or FA 24:0 belong to only part of the lipid classes, not necessarily meaning that these FA would improve endothelial function.

The present study has some limitations.

First, the generalizability of the study might be limited since it was a single-center study with a relatively small sample size. The relatively poor glycemic control (relatively high HbA1c level) of the study population may also lead to limited generalizability. The results should be validated in different populations, such as large populations or those with better glycemic control. Moreover, among the participants in the original study, patients without a measurement of FMD were excluded, and their clinical characteristics were not completely the same as those of the included patients, which might introduce selection bias.

Second, only cross-sectional associations between some lipids and FMD were demonstrated, and it is unclear whether they are causal. Future large-scale prospective studies for validation and additional basic research to elucidate the mechanistic roles of the identified lipids are required.

Third, the same population was used for both the selection and validation of distinctive lipids potentially associated with FMD, although validation using PCAs in an independent population would be desirable.

Fourth, in the PCA based on the data of the lipid selected by the sparse-group LASSO regularized regression analysis, substantial overlaps of principal components between patients with and without endothelial dysfunction were observed. This suggests that the lipids alone have limited discriminatory performance. The relatively small number of patients with endothelial dysfunction in the present study would also be a limitation if the discriminative performance were to be evaluated. The utility of the identified lipids as novel biomarkers should be validated in other larger studies in combination with other clinical parameters.

Fifth, various phosphoinositides derived from the phosphorylation of PI were not assessed in this study. They have distinct cellular functions, and some of these, such as phosphatidylinositol 4,5-bisphosphate and phosphatidylinositol 3,4,5-trisphosphate, are involved in cardiovascular biology and pathology [40]. Assessment of these lipids appears to be informative to the mechanistic understanding of the associations between PIs and endothelial dysfunction.

Nevertheless, the study yielded significant findings that could improve our understanding of the pathophysiology of endothelial dysfunction and help identify potential biomarkers and novel therapeutic targets. Although further validation studies are required, the identified lipids may be useful for the assessment and management of endothelial dysfunction in patients with diabetes.

## 4. Materials and Methods

### 4.1. Study Design and Patients

The present cross-sectional study was conducted as a subanalysis of a previous observational study to investigate the association between plasma metabolites and CAD [22]. In the original study, patients with T2D aged 20–75 years were consecutively recruited at the University of Osaka Hospital and were prospectively observed. The other detailed information was described previously [22]. Of the 240 patients originally enrolled between June 2014 and October 2016, 200 who underwent FMD measurement of the brachial artery were included in the present analysis (Figure 1) and their baseline data was used for the analysis.

The protocol of the original study was approved by the Ethics Committee of the University of Osaka Hospital (approval number: 13454). This study was conducted in accordance with the principles of the Declaration of Helsinki and current legal regulations in Japan. Written informed consent was obtained from all the participants after receiving a full explanation of the study.

### 4.2. Clinical and Biochemical Assessment

The following clinical data were collected from the participants: age, sex, duration of diabetes, smoking status (current smoking or not), BMI, comorbidities, medications, and complications of diabetes. Blood samples were collected after overnight fasting and used for biochemical tests. Total cholesterol, HDL cholesterol, LDL cholesterol, and triglyceride levels were measured using standard techniques. The estimated glomerular filtration rate was calculated according to the guidelines of the Japanese Society of Nephrology [41].

For lipidomic analysis, portions of the blood samples were cooled at 4 °C immediately after collection. The samples were subsequently centrifuged (3000× *g*, 10 min), and the plasma was stored at −80 °C within 4 h.

### 4.3. Assessment of FMD

FMD was assessed using a UNEX EF 38G (UNEX Corporation, Nagoya, Japan) in a quiet, temperature-controlled room in the morning after at least 12 h of fasting, as previously described [22]. To minimize intra- and inter-investigator variations, all measurements were performed by a single expert investigator using a highly reproducible method, as previously reported [42,43,44]. Briefly, the vessel diameter of the brachial artery was measured at rest and after vascularization. The FMD was calculated as follows: FMD (%) = (maximum diameter after avascularization − diameter at rest) × 100/diameter at rest. An FMD value < 4.0% was defined as endothelial dysfunction according to the proposed criteria [23]. This cutoff was proposed by expert consensus based on evidence from studies evaluating the risk of future cardiovascular events and FMD values in patients with established CAD.

### 4.4. Lipidomic Measurement

Semi-targeted plasma lipidomic analysis was performed, as previously described [45,46,47,48]. Briefly, lipidomic measurements were performed using a supercritical fluid chromatography system (Nexera UC system; Shimadzu Corporation, Kyoto, Japan) coupled with a triple quadrupole mass spectrometer (LCMS-8060; Shimadzu Corporation, Kyoto, Japan) operating in the multiple reaction monitoring (MRM) mode. An ACQUITY UPC^2^ Torus diethylamine column (3.0 mm i.d. × 100 mm, 1.7 μm particle size; Waters Corporation, Milford, MA, USA) and an ACQUITY UPC^2^ HSS C18 SB column (3.0 mm i.d. × 100 mm, 1.8 μm particle size, Waters Corporation, Milford, MA, USA) were used for separation.

For lipid extraction, plasma samples were prepared using the Bligh and Dyer method [49] with minor modifications [45,46]. In this procedure, the mixture of labeled lipids from various lipid classes was added as internal standards [45]. A reference sample was prepared by mixing equal amounts (10 µL each) of plasma extracts from the participants. FA constituents of lipid classes were selected using SFC/MS/MS analysis of the hydrolyzed reference sample [47]. Target lipids were subsequently determined by analysis of the reference sample using an in-house MRM library for lipids composed of the selected FA constituents [50]. Targeted quantitative analysis using the selected MRM method [48,50] was then performed on the determined lipids belonging to 16 lipid classes: cholesterol, FA, diacylglycerol (DG), triacylglycerol (TG), cholesterol ester (CE), PC, PE, PI, phosphatidylglycerol (PG), phosphatidylserine (PS), lysophosphatidylcholine (LPC), lysophosphatidylethanolamine (LPE), lysophosphatidylinositol (LPI), ceramide (Cer), HexCer, and sphingomyelin (SM). Data processing was performed using the LabSolutions software (version 5.99 SP2; Shimadzu Corporation, Kyoto, Japan).

### 4.5. Statistical Analysis

Continuous variables are expressed as means and standard deviations or as medians and interquartile ranges, as appropriate. Categorical variables are presented as counts and percentages. A *p*-value < 0.05 was considered statistically significant. All lipid data were log-transformed for statistical analyses.

First, clinical data were compared between patients with and without endothelial dysfunction. Comparisons in the original study were also performed between patients with and without a measurement of FMD. Welch’s *t*-tests and the chi-squared test were used for continuous and categorical variables, respectively, after log-transformation if required. Standardized mean differences are also calculated to assess the magnitude of between-group differences.

Next, a regularized regression model was applied to select the distinctive lipids related to FMD. Because lipids have a group structure known as a lipid class, we used a novel method combining LASSO and group LASSO, called sparse-group LASSO. This method can be fitted to the data with group structures and enables feature selection at both the group and individual levels [51]. The tuning parameter *α*, which indicated the balance between penalties at the group and individual levels, was set to 1/3 [52]. The lipid class, including each lipid species (Table S2), was defined as a group in this analysis. Ten-fold cross-validation was performed to determine the regularization parameter *λ* for the most fitting model to predict FMD. To validate the selection of the distinctive lipids related to FMD, the results of two PCAs were compared. Both analyses were performed using only the lipid data of the participants; one dataset was derived from all the lipids, and the other was from the lipids selected by sparse-group LASSO. Principal components were compared between patients with and without endothelial dysfunction using Welch’s *t*-tests.

Finally, multivariable linear regression analyses were performed to evaluate the association between the selected lipids and FMD. The models were adjusted for the following variables: Model 1 included age and sex, and Model 2 additionally included duration of diabetes, smoking status, BMI, hypertension, HbA1c level, and statin use. To account for multiple testing, we calculated the adjusted *p*-values (*q*-values) with the significance level set at 0.05 using the Benjamini–Hochberg method. This association was also evaluated in a sensitivity analysis restricted to patients without a history of CAD.

Statistical analyses were performed using the R statistical software (version 4.3.1; R Development Core Team, Vienna, Austria). The R package SGPR was used for the sparse-group LASSO. No imputation was performed for missing data.

## 5. Conclusions

This study identified the phospholipids associated with endothelial dysfunction in patients with T2D. The identified lipids included several PE plasmalogens and PIs, and their plasma levels had a positive association with FMD level. These findings suggest that PE plasmalogens and PIs are associated with the prevention of diabetes-accelerated atherosclerosis by inhibiting endothelial dysfunction and may contribute to improving the assessment and management of endothelial dysfunction in patients with diabetes.

## Figures and Tables

**Figure 1:**
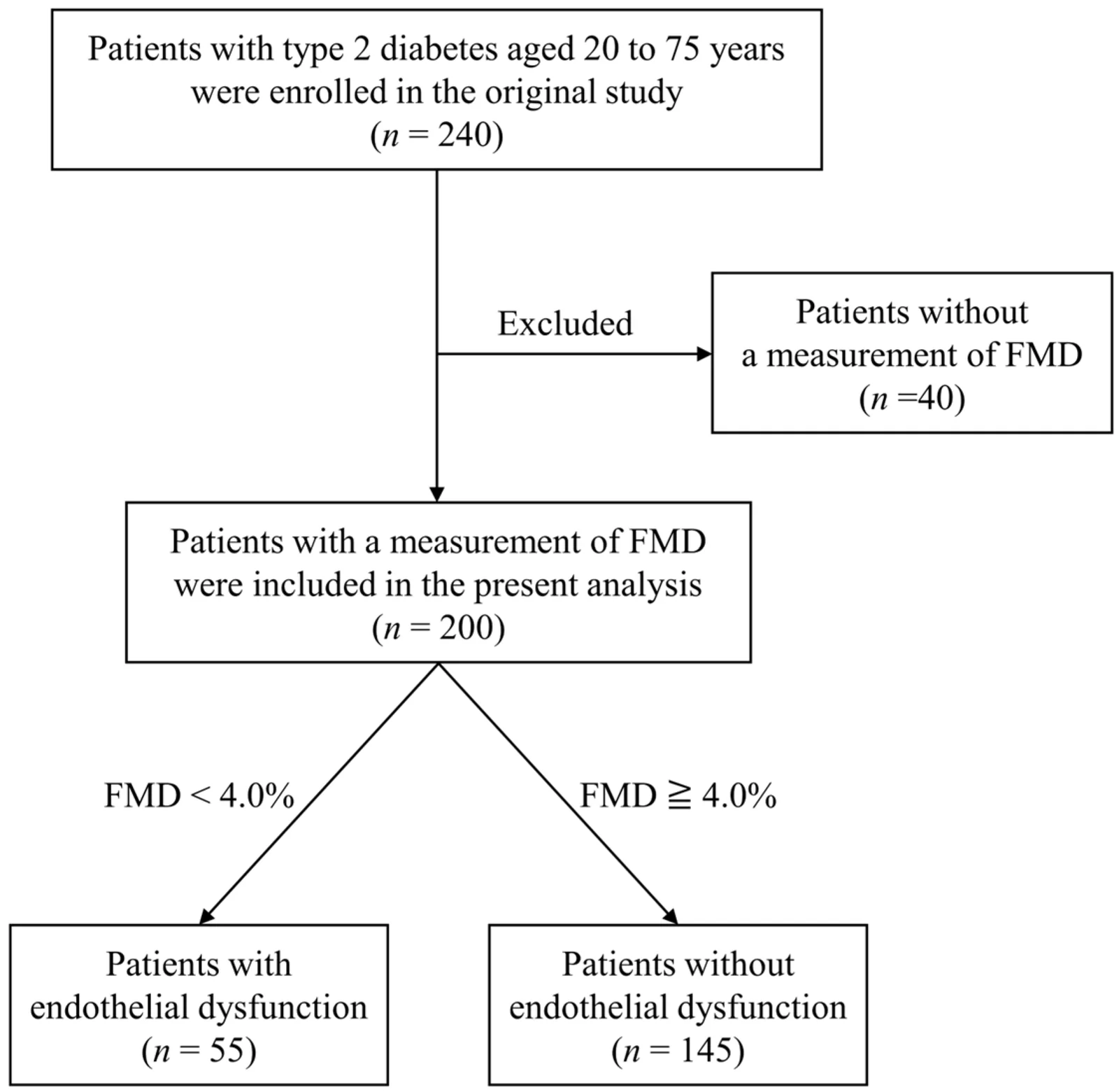
Flowchart of the study population. FMD, flow-mediated vasodilation.

**Figure 2:**
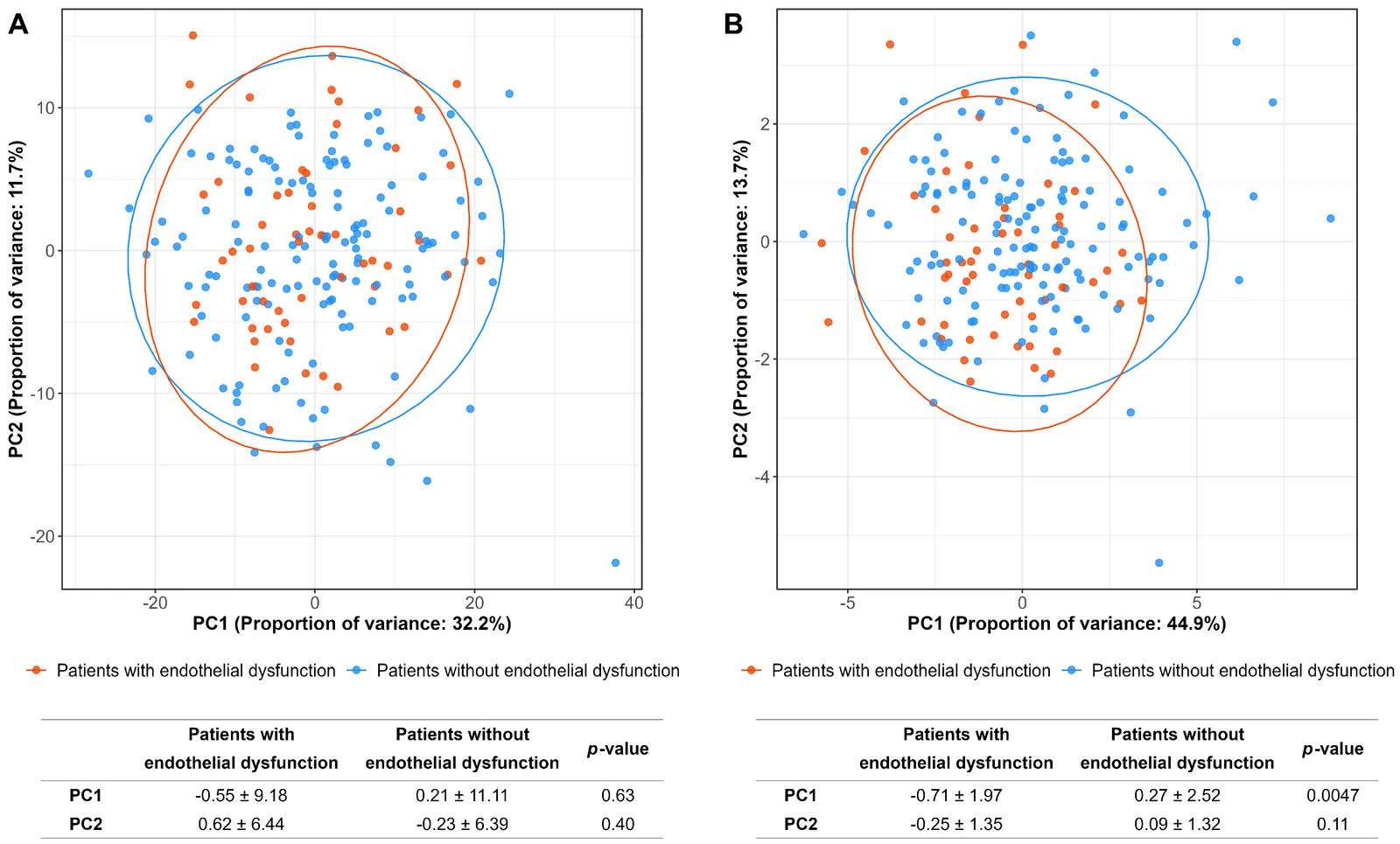
Principal component score plots showing the results of principal component analyses (PCAs). PCAs were performed using the data of all the lipids (**A**) and the lipids selected by the sparse-group LASSO regularized regression model (**B**). Red and blue dots represent patients with and without endothelial dysfunction (FMD  <  4.0%), respectively. Circles indicate 95% prediction ellipses for dots of the same color. Proportion of variance is presented in the axis label. Principal components are presented as means ± standard deviations in the tables and were compared between patients with and without endothelial dysfunction using Welch’s *t*-tests. PC1, first principal component; PC2, second principal component; LASSO, least absolute shrinkage and selection operator; FMD, flow-mediated vasodilation.

**Figure 3:**
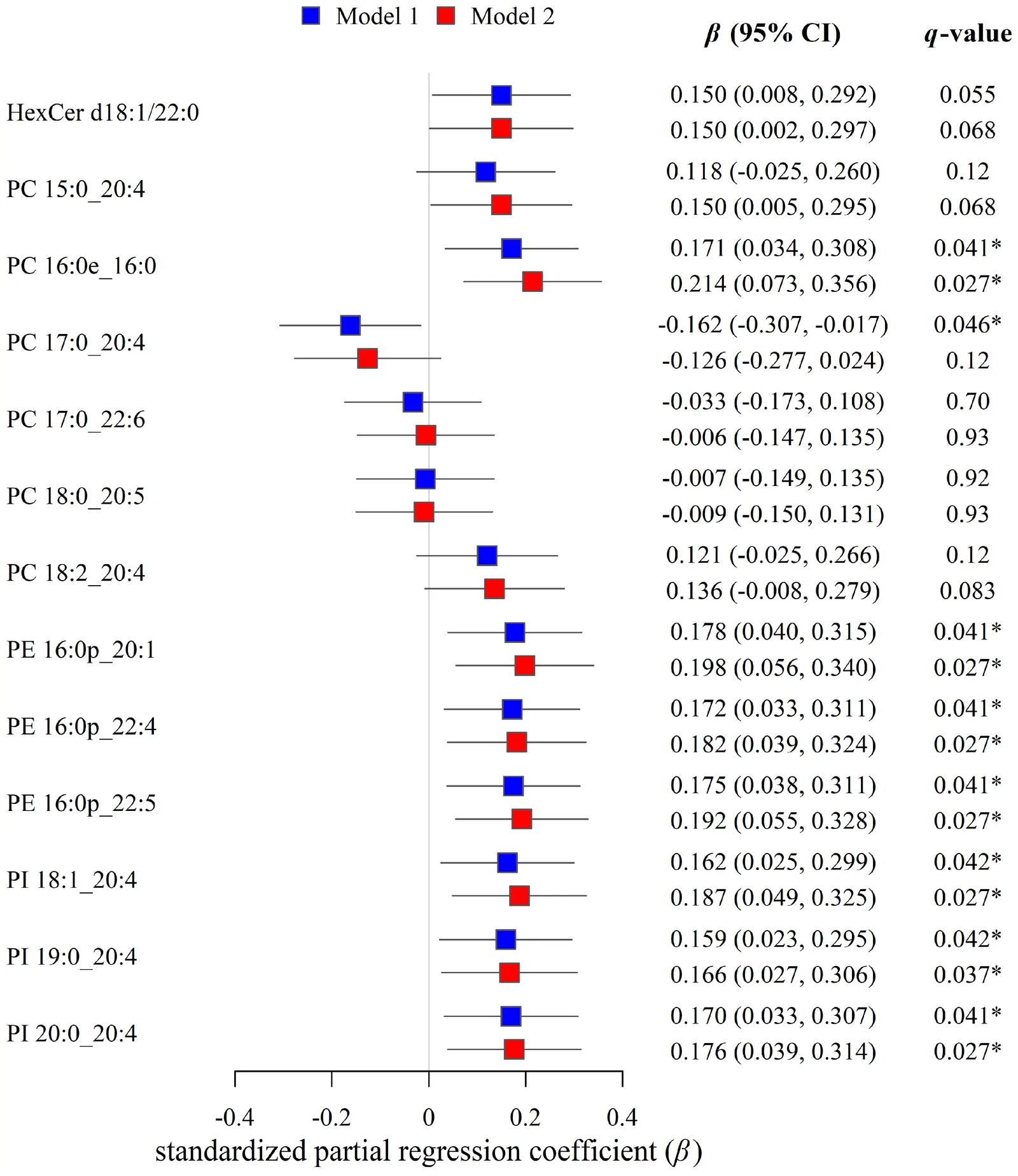
Associations between selected lipids and flow-mediated vasodilation (FMD). Linear regression analysis was performed to evaluate the association between the distinctive lipids selected using the sparse-group LASSO regularized regression model and FMD. Model 1 was adjusted for age and sex. Model 2 was adjusted for duration of diabetes, smoking status, BMI, hypertension, HbA1c level, statin use, and covariates in Model 1. The *q*-values (adjusted *p*-values) were calculated using the Benjamini–Hochberg method to account for multiple testing. * *q*-values indicating statistical significance (<0.05). *β*, standardized partial regression coefficient; 95% CI, 95% confidence interval; HexCer, hexosylceramide; PC, phosphatidylcholine; PE, phosphatidylethanolamine; PI, phosphatidylinositol; LASSO, least absolute shrinkage and selection operator.

**Table 1:** Clinical characteristics of the study participants.

	All (*n* = 200)	Patients with Endothelial Dysfunction (*n* = 55)	Patients Without Endothelial Dysfunction (*n* = 145)	SMD	*p*-Value
Sex: male	110 (55.0)	28 (50.9)	82 (56.6)	0.11	0.58
Age (years)	61.1 ± 11.3	63.4 ± 8.8	60.2 ± 12.0	0.30	0.041
Duration of diabetes (year)	12.6 ± 9.4 *	14.6 ± 11.3 †	11.9 ± 8.5 ‡	0.27	0.11
Current smoking	38 (19.1) §	16 (29.6) †	22 (15.2)	0.35	0.035
BMI (kg/m^2^)	27.53 ± 5.75	28.61 ± 6.10	27.12 ± 5.57	0.25	0.12
Systolic BP (mmHg)	125.3 ± 18.0	126.5 ± 17.2	124.8 ± 18.3	0.09	0.55
Dyslipidemia	133 (66.5)	40 (72.7)	93 (64.1)	0.19	0.33
Hypertension	130 (65.0)	43 (78.2)	87 (60.0)	0.40	0.025
Coronary artery disease	23 (11.5)	6 (10.9)	17 (11.7)	0.03	1.00
Neuropathy	86 (43.7) ||	31 (57.4) †	55 (38.5) ¶	0.39	0.026
Retinopathy	67 (33.7) §	23 (41.8)	44 (30.6) ‡	0.24	0.18
Nephropathy	70 (35.0)	25 (45.5)	45 (31.0)	0.30	0.081
Statin user	86 (43.0)	29 (52.7)	57 (39.3)	0.27	0.12
Total cholesterol (mmol/L)	5.066 ± 1.268	5.001 ± 1.115	5.091 ± 1.324	0.07	0.63
LDL cholesterol (mmol/L)	2.918 ± 0.910	2.938 ± 0.851	2.911 ± 0.934	0.03	0.84
HDL cholesterol (mmol/L)	1.24 ± 0.36	1.17 ± 0.29	1.26 ± 0.39	0.27	0.072
Triglycerides (mmol/L)	1.558 (1.073, 2.509)	1.773 (1.169, 2.405)	1.502 (1.039, 2.506)	0.13	0.40
FPG (mmol/L)	8.436 ± 2.956 #	8.186 ± 2.305 †	8.531 ± 3.170 **	0.12	0.40
HbA1c (%)	9.04 ± 1.79 §	9.19 ± 1.48	8.98 ± 1.90 ‡	0.12	0.41
HbA1c (mmol/mol)	75.3 ± 19.5 §	77.0 ± 16.1	74.6 ± 20.7 ‡	0.07	0.41
eGFR (ml min^−1^ 1.73 m^−2^)	72.89 ± 24.25	68.02 ± 24.63	74.74 ± 23.93	0.07	0.086
u-Alb/Cr (mg/mmol)	1.38 (0.53, 7.01) #	2.66 (0.71, 18.42) †	1.21 (0.46, 4.04) **	0.45	0.0082
Uric acid (μmol/L)	350 ± 92	362 ± 90	345 ± 92	0.18	0.25
FMD (%)	5.77 ± 2.55	2.92 ± 0.82	6.86 ± 2.10	2.46	<0.001

Continuous data are presented as means ± standard deviations or medians (interquartile ranges). Categorical data are presented as counts (percentages). Welch’s *t*-tests and the chi-squared test were used to compare patients with and without endothelial dysfunction (FMD  <  4.0%). * *n* = 198, † *n* = 54, ‡ *n* = 144, § *n* = 199, || *n* = 197, ¶ *n* = 143, # *n* = 196, ** *n* = 142. SMD, standardized mean difference; BP, blood pressure; FPG, fasting plasma glucose; eGFR, estimated glomerular filtration rate; u-Alb/Cr, urine albumin/creatinine ratio; FMD, flow-mediated vasodilation.

## Data Availability

The data presented in this study are not publicly available due to the protocols of the original studies and patient privacy considerations but are available from the corresponding author upon reasonable request.

## Supplementary Materials

The following supporting information can be downloaded at: https://www.mdpi.com/article/10.3390/ijms27198437/s1.

## Abbreviations

The following abbreviations are used in this manuscript:

CVD	Cardiovascular diseases
FMD	Flow-mediated vasodilation
T2D	Type 2 diabetes
CAD	Coronary artery disease
SFC/MS/MS	Supercritical fluid chromatography coupled with tandem mass spectrometry
LASSO	Least absolute shrinkage and selection operator
HexCer	Hexosylceramide
PC	Phosphatidylcholine
PE	Phosphatidylethanolamine
PI	Phosphatidylinositol
PCA	Principal component analysis
FA	Fatty acid
MRM	Multiple reaction monitoring
DG	Diacylglycerol
TG	Triacylglycerol
CE	Cholesterol ester
PG	Phosphatidylglycerol
PS	Phosphatidylserine
LPC	Lysophosphatidylcholine
LPE	Lysophosphatidylethanolamine
LPI	Lysophosphatidylinositol
Cer	Ceramide
SM	Sphingomyelin

## References

[1] Sun H., Saeedi P., Karuranga S., Pinkepank M., Ogurtsova K., Duncan B.B., Stein C., Basit A., Chan J.C.N., Mbanya J.C. (2022). IDF Diabetes Atlas: Global, regional and country-level diabetes prevalence estimates for 2021 and projections for 2045. Diabetes Res. Clin. Pract..

[2] Sarwar N., Gao P., Seshasai S.R., Gobin R., Kaptoge S., Di Angelantonio E., Ingelsson E., Lawlor D.A., Selvin E., Emerging Risk Factors Collaboration (2010). Diabetes mellitus, fasting blood glucose concentration, and risk of vascular disease: A collaborative meta-analysis of 102 prospective studies. Lancet.

[3] Wong N.D., Sattar N. (2023). Cardiovascular risk in diabetes mellitus: Epidemiology, assessment and prevention. Nat. Rev. Cardiol..

[4] Wang C.C.L., Hess C.N., Hiatt W.R., Goldfine A.B. (2016). Clinical update: Cardiovascular disease in diabetes mellitus: Atherosclerotic cardiovascular disease and heart failure in type 2 diabetes mellitus—Mechanisms, management, and clinical considerations. Circulation.

[5] Khan A.W., Jandeleit-Dahm K.A.M. (2025). Atherosclerosis in diabetes mellitus: Novel mechanisms and mechanism-based therapeutic approaches. Nat. Rev. Cardiol..

[6] Pasut A., Lama E., Van Craenenbroeck A.H., Kroon J., Carmeliet P. (2025). Endothelial cell metabolism in cardiovascular physiology and disease. Nat. Rev. Cardiol..

[7] Dhananjayan R., Koundinya K.S., Malati T., Kutala V.K. (2016). Endothelial dysfunction in type 2 diabetes mellitus. Indian J. Clin. Biochem..

[8] Teragawa H., Kato M., Kurokawa J., Yamagata T., Matsuura H., Chayama K. (2001). Usefulness of flow-mediated dilation of the brachial artery and/or the intima-media thickness of the carotid artery in predicting coronary narrowing in patients suspected of having coronary artery disease. Am. J. Cardiol..

[9] Thijssen D.H.J., Bruno R.M., van Mil A.C.C.M., Holder S.M., Faita F., Greyling A., Zock P.L., Taddei S., Deanfield J.E., Luscher T. (2019). Expert consensus and evidence-based recommendations for the assessment of flow-mediated dilation in humans. Eur. Heart J..

[10] Deanfield J.E., Halcox J.P., Rabelink J.T. (2007). Endothelial function and dysfunction: Testing and clinical relevance. Circulation.

[11] Lupattelli G., Lombardini R., Schillaci G., Ciuffetti G., Marchesi S., Siepi D., Mannarino E. (2000). Flow-mediated vasoactivity and circulating adhesion molecules in hypertriglyceridemia: Association with small, dense LDL cholesterol particles. Am. Heart J..

[12] Yunoki K., Nakamura K., Miyoshi T., Enko K., Kohno K., Morita H., Kusano K.F., Ito H. (2011). Ezetimibe improves postprandial hyperlipemia and its induced endothelial dysfunction. Atherosclerosis.

[13] Salihovic S., Lamichane S., Hyötyläinen T., Orešič M. (2023). Recent advances towards mass spectrometry-based clinical lipidomics. Curr. Opin. Chem. Biol..

[14] McGranaghan P., Kirwan J.A., Garcia-Rivera M.A., Pieske B., Edelmann F., Blaschke F., Appunni S., Saxena A., Rubens M., Veledar E. (2021). Lipid metabolite biomarkers in cardiovascular disease: Discovery and biomechanism translation from human studies. Metabolites.

[15] Kale D., Fatangare A., Phapale P., Sickmann A. (2023). Blood-derived lipid and metabolite biomarkers in cardiovascular research from clinical studies: A recent update. Cells.

[16] Surendran A., Zhang H., Stamenkovic A., Ravandi A. (2025). Lipidomics and cardiovascular disease. Biochim. Biophys. Acta (BBA)—Mol. Basis Dis..

[17] Havulinna A.S., Sysi-Aho M., Hilvo M., Kauhanen D., Hurme R., Ekroos K., Salomaa V., Laaksonen R. (2016). Circulating ceramides predict cardiovascular outcomes in the population-based FINRISK 2002 cohort. Arterioscler. Thromb. Vasc. Biol..

[18] Laaksonen R., Ekroos K., Sysi-Aho M., Hilvo M., Vihervaara T., Kauhanen D., Suoniemi M., Hurme R., März W., Scharnagl H. (2016). Plasma ceramides predict cardiovascular death in patients with stable coronary artery disease and acute coronary syndromes beyond LDL-cholesterol. Eur. Heart J..

[19] Sigruener A., Kleber M.E., Heimerl S., Liebisch G., Schmitz G., Maerz W. (2014). Glycerophospholipid and sphingolipid species and mortality: The Ludwigshafen Risk and cardiovascular Health (LURIC) study. PLoS ONE.

[20] Klingenberg R., Leiherer A., Dobrev D., Kaski J.C., Levkau B., März W., Sossalla S., von Eckardstein A., Drexel H. (2025). Ceramides in cardiovascular disease: Emerging role as independent risk predictors and novel therapeutic targets. Cardiovasc. Res..

[21] Chen J., Cheng Y., Cai X., Wang M., Zhang Z., Wei S., Zhou S., Chen Y. (2026). Association between plasma ceramide levels and endothelial dysfunction in patients with acute coronary syndrome: A prospective observational study. Medicine.

[22] Omori K., Katakami N., Arakawa S., Yamamoto Y., Ninomiya H., Takahara M., Matsuoka T.A., Tsugawa H., Furuno M., Bamba T. (2020). Identification of plasma inositol and indoxyl sulfate as novel biomarker candidates for atherosclerosis in patients with type 2 diabetes. -Findings from Metabolome Analysis Using GC/MS-. J. Atheroscler. Thromb..

[23] Tanaka A., Tomiyama H., Maruhashi T., Matsuzawa Y., Miyoshi T., Kabutoya T., Kario K., Sugiyama S., Munakata M., Ito H. (2018). Physiological Diagnosis Criteria for Vascular Failure Committee. Physiological diagnostic criteria for vascular failure. Hypertension.

[24] Kaur R., Kaur M., Singh J. (2018). Endothelial dysfunction and platelet hyperactivity in type 2 diabetes mellitus: Molecular insights and therapeutic strategies. Cardiovasc. Diabetol..

[25] Morita S.Y., Nakano M., Sakurai A., Deharu Y., Vertut-Doi A., Handa T. (2005). Formation of ceramide-enriched domains in lipid particles enhances the binding of apolipoprotein E. FEBS Lett..

[26] Morita S.Y., Okuhira K., Tsuchimoto N., Vertut-Doï A., Saito H., Nakano M., Handa T. (2003). Effects of sphingomyelin on apolipoprotein E- and lipoprotein lipase-mediated cell uptake of lipid particles. Biochim. Biophys. Acta (BBA)—Mol. Cell Biol. Lipids.

[27] Morita S.Y. (2024). Phospholipid biomarkers of coronary heart disease. J. Pharm. Health Care Sci..

[28] Hahnel D., Thiery J., Brosche T., Engelmann B. (1999). Role of plasmalogens in the enhanced resistance of LDL to copper-induced oxidation after LDL apheresis. Arter. Thromb. Vasc. Biol..

[29] Maeba R., Ueta N. (2003). Ethanolamine plasmalogens prevent the oxidation of cholesterol by reducing the oxidizability of cholesterol in phospholipid bilayers. J. Lipid Res..

[30] Maeba R., Maeda T., Kinoshita M., Takao K., Takenaka H., Kusano J., Yoshimura N., Takeoka Y., Yasuda D., Okazaki T. (2007). Plasmalogens in human serum positively correlate with high-density lipoprotein and decrease with aging. J. Atheroscler. Thromb..

[31] Meikle P.J., Wong G., Tsorotes D., Barlow C.K., Weir J.M., Christopher M.J., MacIntosh G.L., Goudey B., Stern L., Kowalczyk A. (2011). Plasma lipidomic analysis of stable and unstable coronary artery disease. Arterioscler. Thromb. Vasc. Biol..

[32] Saeed A., McKennan C., Duan J., Yang Y.N., Kip K.E., Finegold D., Vu M., Swanson J., Lopez O.L., Cohen A. (2025). Mid-life anti-inflammatory metabolites are inversely associated with long-term cardiovascular disease events. eBioMedicine.

[33] Huang Y., Mu R., Wen D., Grimsby J.S., Liang M., Rosenbaum A.I. (2021). Differences in levels of phosphatidylinositols in healthy and stable coronary artery disease subjects revealed by HILIC-MRM method with SERRF normalization. PLoS ONE.

[34] Zhao Y., Qian Y., Sun Z., Shen X., Cai Y., Li L., Wang Z. (2021). Role of PI3K in the progression and regression of atherosclerosis. Front. Pharmacol..

[35] Zhang Y., Han L., Wang Y., Wang M. (2025). The role of the PI3K/Akt/mTOR pathway in atherosclerosis: Mechanisms, therapeutic potential, and emerging targeted treatments. Curr. Atheroscler. Rep..

[36] Annevelink C.E., Sapp P.A., Petersen K.S., Shearer G.C., Kris-Etherton P.M. (2023). Diet-derived and diet-related endogenously produced palmitic acid: Effects on metabolic regulation and cardiovascular disease risk. J. Clin. Lipidol..

[37] Wang B., Wu L., Chen J., Dong L., Chen C., Wen Z., Hu J., Fleming I., Wang D.W. (2021). Metabolism pathways of arachidonic acids: Mechanisms and potential therapeutic targets. Signal Transduct. Target. Ther..

[38] Hu Y., Li W., Cheng X., Yang H., She Z.G., Cai J., Li H., Zhang X.J. (2024). Emerging roles and therapeutic applications of arachidonic acid pathways in cardiometabolic diseases. Circ. Res..

[39] Marklund M., Wu J.H.Y., Imamura F., Del Gobbo L.C., Fretts A., de Goede J., Shi P., Tintle N., Wennberg M., Aslibekyan S. (2019). Biomarkers of dietary omega-6 fatty acids and incident cardiovascular disease and mortality: An individual-level pooled analysis of 30 cohort studies. Circulation.

[40] Krajnik A., Brazzo J.A., Vaidyanathan K., Das T., Redondo-Muñoz J., Bae Y. (2020). Phosphoinositide signaling and mechanotransduction in cardiovascular biology and disease. Front. Cell Dev. Biol..

[41] Matsuo S., Imai E., Horio M., Yasuda Y., Tomita K., Nitta K., Yamagata K., Tomino Y., Yokoyama H., Hishida A. (2009). Revised equations for estimated GFR from serum creatinine in Japan. Am. J. Kidney Dis..

[42] Corretti M.C., Anderson T.J., Benjamin E.J., Celermajer D., Charbonneau F., Creager M.A., Deanfield J., Drexler H., Gerhard-Herman M., Herrington D. (2002). International Brachial Artery Reactivity Task Force. Guidelines for the ultrasound assessment of endothelial-dependent flow-mediated vasodilation of the brachial artery: A report of the International Brachial Artery Reactivity Task Force. J. Am. Coll. Cardiol..

[43] Tomiyama H., Higashi Y., Takase B., Node K., Sata M., Inoue T., Ishibashi Y., Ueda S., Shimada K., Yamashina A. (2011). Relationships among hyperuricemia, metabolic syndrome, and endothelial function. Am. J. Hypertens..

[44] Ninomiya H., Katakami N., Sato I., Osawa S., Yamamoto Y., Takahara M., Kawamori D., Matsuoka T.A., Shimomura I. (2018). Association between subclinical atherosclerosis markers and the level of accumulated advanced glycation end-products in the skin of patients with diabetes. J. Atheroscler. Thromb..

[45] Taya N., Katakami N., Omori K., Hosoe S., Watanabe H., Takahara M., Miyashita K., Konya Y., Obara S., Hidaka A. (2025). Plasma diacylglycerols are associated with carotid intima-media thickness among patients with type 2 diabetes: Findings from a supercritical fluid chromatography/mass spectrometry-based semi-targeted lipidomic analysis. Int. J. Mol. Sci..

[46] Taya N., Katakami N., Omori K., Hosoe S., Watanabe H., Takahara M., Miyashita K., Nishizawa H., Konya Y., Obara S. (2023). Change in fatty acid composition of plasma triglyceride caused by a 2 week comprehensive risk management for diabetes: A prospective observational study of type 2 diabetes patients with supercritical fluid chromatography/mass spectrometry-based semi-target lipidomic analysis. J. Diabetes Investig..

[47] Ogawa T., Izumi Y., Kusumoto K., Fukusaki E., Bamba T. (2017). Wide target analysis of acylglycerols in miso (Japanese fermented soybean paste) by supercritical fluid chromatography coupled with triple quadrupole mass spectrometry and the analysis of the correlation between taste and both acylglycerols and free fatty acids. Rapid Commun. Mass Spectrom..

[48] Fushimi T., Izumi Y., Takahashi M., Hata K., Murano Y., Bamba T. (2020). Dynamic metabolome analysis reveals the metabolic fate of medium-chain fatty acids in AML12 cells. J. Agric. Food Chem..

[49] Bligh E.G., Dyer W.J. (1959). A rapid method of total lipid extraction and purification. Can. J. Biochem. Physiol..

[50] Takeda H., Izumi Y., Takahashi M., Paxton T., Tamura S., Koike T., Yu Y., Kato N., Nagase K., Shiomi M. (2018). Widely-targeted quantitative lipidomics method by supercritical fluid chromatography triple quadrupole mass spectrometry. J. Lipid Res..

[51] Simon N., Friedman J., Hastie T., Tibshirani R. (2013). A sparse-group lasso. J. Comput. Graph. Stat..

[52] Buch G., Schulz A., Schmidtmann I., Strauch K., Wild P.S. (2024). Sparse group penalties for bi-level variable selection. Biom. J..

