# Supplementary material for: Plasma Lipids Associated with Endothelial Dysfunction in Patients with Type 2 Diabetes: A Cross-Sectional Study Using Lipidomic Analysis
Source: Int J Mol Sci. 2026 Sep 22;27(19):8437. doi: 10.3390/ijms27198437 (PMC13665545; doi:10.3390/ijms27198437)
Supplement: Supplementary file 1 [file ijms-27-08437-s001.zip › ijms-4557476-supplementary.pdf]

Supplementary Materials

Plasma Lipids Associated with Endothelial Dysfunction in Patients with Type 2 Diabetes: A Cross-Sectional Study Using Lipidomic Analysis

Table S1. Clinical characteristics of the participants of the original study

Table S2. List of all identified lipids and their lipid class

Figure S1. The results of the selection of the distinctive lipids related to flow-mediated vasodilation (FMD) using sparse-group LASSO regularized regression model

Figure S2. Associations between selected lipids and flow-mediated vasodilation (FMD) in patients without a history of coronary artery disease

Table S1. Clinical characteristics of the participants of the original study

	All (n = 240)	Patients with a measurement of FMD (n = 200)	Patients without a measurement of FMD (n = 40)	SMD	p-value
Sex: male	140 (58.3)	110 (55.0)	30 (75.0)	0.43	0.030
Age (years)	60.6 ± 11.5	61.1 ± 11.3	58.2 ± 12.3	0.25	0.17
Duration of diabetes (year)	13.1 ± 9.8 *	12.6 ± 9.4 †	15.7 ± 11.7 ‡	0.29	0.13
Current smoking	47 (19.9) §	38 (19.1) ‖	9 (24.3) ¶	0.13	0.61
BMI (kg/m^2)	27.50 ± 5.84 *	27.53 ± 5.75	27.32 ± 6.39 ¶	0.04	0.85
Systolic BP (mmHg)	125.0 ± 17.8 #	125.3 ± 18.0	123.5 ± 17.0 **	0.10	0.56
Dyslipidemia	158 (65.8)	133 (66.5)	25 (62.5)	0.08	0.76
Hypertension	156 (65.0)	130 (65.0)	26 (65.0)	0.00	1.00
Coronary artery disease	40 (16.7)	23 (11.5)	17 (42.5)	0.75	<0.001
Neuropathy	111 (47.6) ††	86 (43.7) ‡‡	25 (69.4) §§	0.54	0.0076
Retinopathy	81 (34.5) ‖‖	67 (33.7) ‖	14 (38.9) §§	0.11	0.68
Nephropathy	85 (35.7) #	70 (35.0)	15 (39.5) **	0.09	0.73
Statin user	105 (43.8)	86 (43.0)	19 (47.5)	0.09	0.73
Total cholesterol (mmol/L)	5.064 ± 1.282	5.066 ± 1.268	5.054 ± 1.363	0.01	0.96
LDL cholesterol (mmol/L)	2.934 ± 0.979	2.918 ± 0.910	3.011 ± 1.281	0.08	0.66
HDL cholesterol (mmol/L)	1.25 ± 0.38	1.24 ± 0.36	1.28 ± 0.43	0.11	0.55
Triglycerides (mmol/L)	1.536 (1.058, 2.481)	1.558 (1.073, 2.509)	1.394 (0.909, 2.317)	0.21	0.24
FPG (mmol/L)	8.460 ± 2.861 §	8.436 ± 2.956 ¶¶	8.579 ± 2.370	0.05	0.74
HbA1c (%)	9.00 ± 1.80 ##	9.04 ± 1.79 ‖	8.82 ± 1.85	0.12	0.50
HbA1c (mmol/mol)	74.9 ± 19.6 ##	75.3 ± 19.5 ‖	72.9 ± 20.2	0.04	0.84

eGFR (ml min^{-1} 1.73 m^{-2})	72.82 ± 24.17	72.89 ± 24.25	72.48 ± 24.07	0.02	0.92
u-Alb/Cr (mg/mmol)	1.39 (0.55, 7.26) ||||	1.38 (0.53, 7.01) ¶¶	1.54 (0.64, 12.18) ‡	0.15	0.38
Uric acid (μmol/L)	355 ± 92	350 ± 92	382 ± 89	0.36	0.041

Continuous data are presented as means ± standard deviations or medians (interquartile ranges). Categorical data are presented as counts (percentages). Welch's *t*-tests and the chi-squared test were used to compare patients with and without a measurement of FMD.

**n* = 237, †*n* = 198, ‡*n* = 39, §*n* = 236, ||*n* = 199, ¶*n* = 37, #*n* = 238, ***n* = 38, ††*n* = 233, ‡‡*n* = 197, §§*n* = 36, ||||*n* = 235, ¶¶*n* = 196, ##*n* = 239.

FMD, flow-mediated vasodilation; SMD, standardized mean difference; BP, blood pressure; FPG, fasting plasma glucose; eGFR, estimated glomerular filtration rate; u-Alb/Cr, urine albumin/creatinine ratio.

Table S2. List of all identified lipids and their lipid class

Lipid specie	Lipid class
FFA 12:0	FFA
FFA 14:0	FFA
FFA 14:1	FFA
FFA 16:0	FFA
FFA 16:1	FFA
FFA 17:0	FFA
FFA 17:1	FFA
FFA 18:0	FFA
FFA 18:1	FFA
FFA 18:2	FFA
FFA 18:3	FFA
FFA 19:0	FFA
FFA 19:1	FFA
FFA 20:0	FFA
FFA 20:1	FFA
FFA 20:2	FFA
FFA 20:3	FFA
FFA 20:4	FFA
FFA 20:5	FFA
FFA 22:1	FFA
FFA 22:2	FFA
FFA 22:3	FFA
FFA 22:4	FFA
FFA 22:5	FFA
FFA 22:6	FFA
FFA 23:0	FFA
FFA 24:0	FFA
FFA 24:1	FFA
DG 12:0_18:1	DG
DG 12:0_18:2	DG
DG 14:0_16:0	DG
DG 14:0_18:1	DG
DG 14:0_18:2	DG
DG 16:0_16:0	DG
DG 16:0_16:1	DG

DG 16:0_18:0	DG
DG 16:0_18:1	DG
DG 16:0_18:2	DG
DG 16:0_18:3	DG
DG 16:0_20:4	DG
DG 16:0_20:5	DG
DG 16:1_18:1	DG
DG 16:1_18:2	DG
DG 17:1_18:2	DG
DG 18:0_18:1	DG
DG 18:1_18:1	DG
DG 18:1_18:2	DG
DG 18:1_18:3	DG
DG 18:1_20:3	DG
DG 18:1_20:4	DG
DG 18:1_20:5	DG
DG 18:2_18:2	DG
DG 18:2_18:3	DG
DG 18:2_20:4	DG
TG 14:0_14:0_14:0	TG
TG 14:0_14:0_16:0	TG
TG 14:0_14:0_16:1	TG
TG 14:0_14:0_18:0	TG
TG 14:0_14:0_18:1	TG
TG 14:0_14:0_18:2	TG
TG 14:0_16:0_16:0	TG
TG 14:0_16:0_16:1	TG
TG 14:0_16:0_18:0	TG
TG 14:0_16:0_18:1	TG
TG 14:0_16:0_18:2	TG
TG 14:0_16:0_20:4	TG
TG 14:0_16:0_20:5	TG
TG 14:0_16:0_22:6	TG
TG 14:0_16:1_16:1	TG
TG 14:0_16:1_18:0	TG
TG 14:0_16:1_18:1	TG
TG 14:0_16:1_18:2	TG

TG 14:0_18:1_18:1	TG
TG 14:0_18:1_18:2	TG
TG 14:0_18:2_18:2	TG
TG 14:0_20:4_20:4	TG
TG 16:0_16:0_16:0	TG
TG 16:0_16:0_16:1	TG
TG 16:0_16:0_18:0	TG
TG 16:0_16:0_18:1	TG
TG 16:0_16:0_18:2	TG
TG 16:0_16:0_22:6	TG
TG 16:0_16:1_16:1	TG
TG 16:0_16:1_18:0	TG
TG 16:0_16:1_18:1	TG
TG 16:0_16:1_18:2	TG
TG 16:0_16:1_20:3	TG
TG 16:0_16:1_20:5	TG
TG 16:0_16:1_22:5	TG
TG 16:0_16:1_22:6	TG
TG 16:0_18:0_18:0	TG
TG 16:0_18:0_18:1	TG
TG 16:0_18:0_18:2	TG
TG 16:0_18:1_18:1	TG
TG 16:0_18:1_18:2	TG
TG 16:0_18:1_20:1	TG
TG 16:0_18:1_20:4	TG
TG 16:0_18:1_20:5	TG
TG 16:0_18:1_22:6	TG
TG 16:0_18:2_18:2	TG
TG 16:0_18:2_20:0	TG
TG 16:0_18:2_20:1	TG
TG 16:0_18:2_20:2	TG
TG 16:0_18:2_20:3	TG
TG 16:0_18:2_20:4	TG
TG 16:0_18:2_20:5	TG
TG 16:0_18:2_22:1	TG
TG 16:0_18:2_22:5	TG
TG 16:1_16:1_16:1	TG

TG 16:1_16:1_18:1	TG
TG 16:1_16:1_18:2	TG
TG 16:1_16:1_20:4	TG
TG 16:1_16:1_20:5	TG
TG 16:1_16:1_22:5	TG
TG 16:1_16:1_22:6	TG
TG 16:1_18:1_18:1	TG
TG 16:1_18:1_18:2	TG
TG 16:1_18:1_20:5	TG
TG 16:1_18:1_22:6	TG
TG 16:1_18:2_18:2	TG
TG 16:1_18:2_20:4	TG
TG 16:1_18:2_20:5	TG
TG 16:1_18:2_22:5	TG
TG 16:1_18:2_22:6	TG
TG 18:0_18:0_18:1	TG
TG 18:0_18:1_18:1	TG
TG 18:0_18:1_20:4	TG
TG 18:1_18:1_18:1	TG
TG 18:1_18:1_18:2	TG
TG 18:1_18:1_20:4	TG
TG 18:1_18:1_20:5	TG
TG 18:1_18:1_22:5	TG
TG 18:1_18:1_22:6	TG
TG 18:1_18:2_18:2	TG
TG 18:1_18:2_20:2	TG
TG 18:1_18:2_20:5	TG
TG 18:1_18:2_22:5	TG
TG 18:1_18:2_22:6	TG
TG 18:2_18:2_18:2	TG
TG 18:2_18:2_20:1	TG
TG 18:2_18:2_20:5	TG
TG 18:2_18:2_22:5	TG
TG 18:2_20:4_20:4	TG
CE 16:1	CE
CE 18:1	CE
CE 18:2	CE

CE 18:3	CE
CE 20:1	CE
CE 20:2	CE
CE 20:3	CE
CE 20:4	CE
CE 20:5	CE
CE 22:5	CE
CE 22:6	CE
Cholesterol	Cholesterol
PC 14:0_16:0	PC
PC 14:0_18:2	PC
PC 15:0_18:1	PC
PC 15:0_18:2	PC
PC 15:0_20:3	PC
PC 15:0_20:4	PC
PC 16:0_16:0	PC
PC 16:0_16:1	PC
PC 16:0_17:0	PC
PC 16:0_18:0	PC
PC 16:0_18:1	PC
PC 16:0_18:2	PC
PC 16:0_18:3	PC
PC 16:0_20:1	PC
PC 16:0_20:2	PC
PC 16:0_20:3	PC
PC 16:0_20:4	PC
PC 16:0_20:5	PC
PC 16:0_22:2	PC
PC 16:0_22:4	PC
PC 16:0_22:5	PC
PC 16:0_22:6	PC
PC 16:0e_16:0	PC
PC 16:0e_18:1	PC
PC 16:0e_18:2	PC
PC 16:1_18:0	PC
PC 16:1_18:1	PC
PC 16:1_18:2	PC

PC 16:1_22:6	PC
PC 17:0_18:1	PC
PC 17:0_18:2	PC
PC 17:0_20:4	PC
PC 17:0_22:6	PC
PC 18:0_18:1	PC
PC 18:0_18:2	PC
PC 18:0_20:2	PC
PC 18:0_20:3	PC
PC 18:0_20:4	PC
PC 18:0_20:5	PC
PC 18:0_22:5	PC
PC 18:0_22:6	PC
PC 18:0e_18:1	PC
PC 18:0e_18:2	PC
PC 18:1_18:1	PC
PC 18:1_18:2	PC
PC 18:1_18:3	PC
PC 18:1_20:1	PC
PC 18:1_20:2	PC
PC 18:1_20:3	PC
PC 18:1_20:4	PC
PC 18:1_20:5	PC
PC 18:1_22:5	PC
PC 18:1_22:6	PC
PC 18:2_18:2	PC
PC 18:2_18:3	PC
PC 18:2_20:1	PC
PC 18:2_20:2	PC
PC 18:2_20:3	PC
PC 18:2_20:4	PC
PC 18:2_20:5	PC
PC 18:2_22:6	PC
PE 16:0_18:0	PE
PE 16:0_18:1	PE
PE 16:0_18:2	PE
PE 16:0_18:3	PE

PE 16:0_20:3	PE
PE 16:0_20:4	PE
PE 16:0_20:5	PE
PE 16:0p_20:1	PE
PE 16:0p_20:2	PE
PE 16:0p_20:4	PE
PE 16:0p_22:4	PE
PE 16:0p_22:5	PE
PE 16:0p_22:6	PE
PE 17:0_18:2	PE
PE 17:0_20:4	PE
PE 18:0_18:1	PE
PE 18:0_18:2	PE
PE 18:0_18:3	PE
PE 18:0_20:3	PE
PE 18:0_20:4	PE
PE 18:0_20:5	PE
PE 18:0p_20:4	PE
PE 18:0p_20:5	PE
PE 18:1_18:1	PE
PE 18:1_18:2	PE
PE 18:1_18:3	PE
PE 18:1_20:3	PE
PE 18:1_20:4	PE
PE 18:1_20:5	PE
PE 18:1p_20:5	PE
PE 18:1p_22:5	PE
PE 18:1p_22:6	PE
PE 18:2_20:4	PE
PI 16:0_16:1	PI
PI 16:0_18:0	PI
PI 16:0_18:1	PI
PI 16:0_18:2	PI
PI 16:0_20:2	PI
PI 16:0_20:3	PI
PI 16:0_20:4	PI
PI 16:0_22:5	PI

PI 16:0_22:6	PI
PI 16:1_18:0	PI
PI 16:1_18:1	PI
PI 17:0_18:1	PI
PI 17:0_18:2	PI
PI 17:0_20:4	PI
PI 18:0_18:0	PI
PI 18:0_18:1	PI
PI 18:0_18:2	PI
PI 18:0_18:3	PI
PI 18:0_20:2	PI
PI 18:0_20:3	PI
PI 18:0_20:4	PI
PI 18:0_20:5	PI
PI 18:0_22:4	PI
PI 18:0_22:5	PI
PI 18:0_22:6	PI
PI 18:1_18:1	PI
PI 18:1_18:2	PI
PI 18:1_20:3	PI
PI 18:1_20:4	PI
PI 19:0_20:4	PI
PI 20:0_20:4	PI
PG 16:0_18:1	PG
PG 18:0_18:1	PG
PG 18:0_18:2	PG
PS 18:0_18:1	PS
LPC 14:0	LPC
LPC 15:0	LPC
LPC 16:0	LPC
LPC 16:1	LPC
LPC 17:0	LPC
LPC 17:1	LPC
LPC 18:0	LPC
LPC 18:1	LPC
LPC 18:2	LPC
LPC 18:3	LPC

LPC 19:0	LPC
LPC 19:1	LPC
LPC 20:0	LPC
LPC 20:1	LPC
LPC 20:2	LPC
LPC 20:3	LPC
LPC 20:4	LPC
LPC 20:5	LPC
LPC 22:0	LPC
LPC 22:1	LPC
LPC 22:2	LPC
LPC 22:4	LPC
LPC 22:5	LPC
LPC 22:6	LPC
LPC 23:0	LPC
LPC 24:0	LPC
LPC 25:0	LPC
LPC 26:0	LPC
LPC 27:0	LPC
LPE 16:0	LPE
LPE 18:0	LPE
LPE 18:1	LPE
LPE 18:2	LPE
LPE 20:4	LPE
LPE 22:6	LPE
LPI 18:0	LPI
LPI 18:1	LPI
LPI 20:4	LPI
Cer d18:1/22:2	Cer
Cer d18:1/24:0	Cer
Cer t18:0/20:4	Cer
Cer t18:0/22:4	Cer
Cer t18:0/22:5	Cer
HexCer d18:1/16:0	HexCer
HexCer d18:1/18:0	HexCer
HexCer d18:1/20:0	HexCer
HexCer d18:1/22:0	HexCer

HexCer d18:1/23:0	HexCer
HexCer d18:1/24:0	HexCer
HexCer d18:1/24:1	HexCer
SM d18:1/12:0	SM
SM d18:1/14:0	SM
SM d18:1/14:1	SM
SM d18:1/15:0	SM
SM d18:1/16:0	SM
SM d18:1/16:1	SM
SM d18:1/17:0	SM
SM d18:1/17:1	SM
SM d18:1/18:0	SM
SM d18:1/18:1	SM
SM d18:1/18:2	SM
SM d18:1/20:1	SM
SM d18:1/24:0	SM
SM d18:1/24:1	SM
SM d18:1/25:0	SM

FFA, free fatty acid; DG, diacylglycerol; TG, triacylglycerol; CE, cholesterol ester; PC, phosphatidylcholine; PE, phosphatidylethanolamine; PI, phosphatidylinositol; PG, phosphatidylglycerol; PS, phosphatidylserine; LPC, lysophosphatidylcholine; LPE, lysophosphatidylethanolamine; LPI, lysophosphatidylinositol; Cer, ceramide; HexCer, hexosylceramide; SM, sphingomyelin.

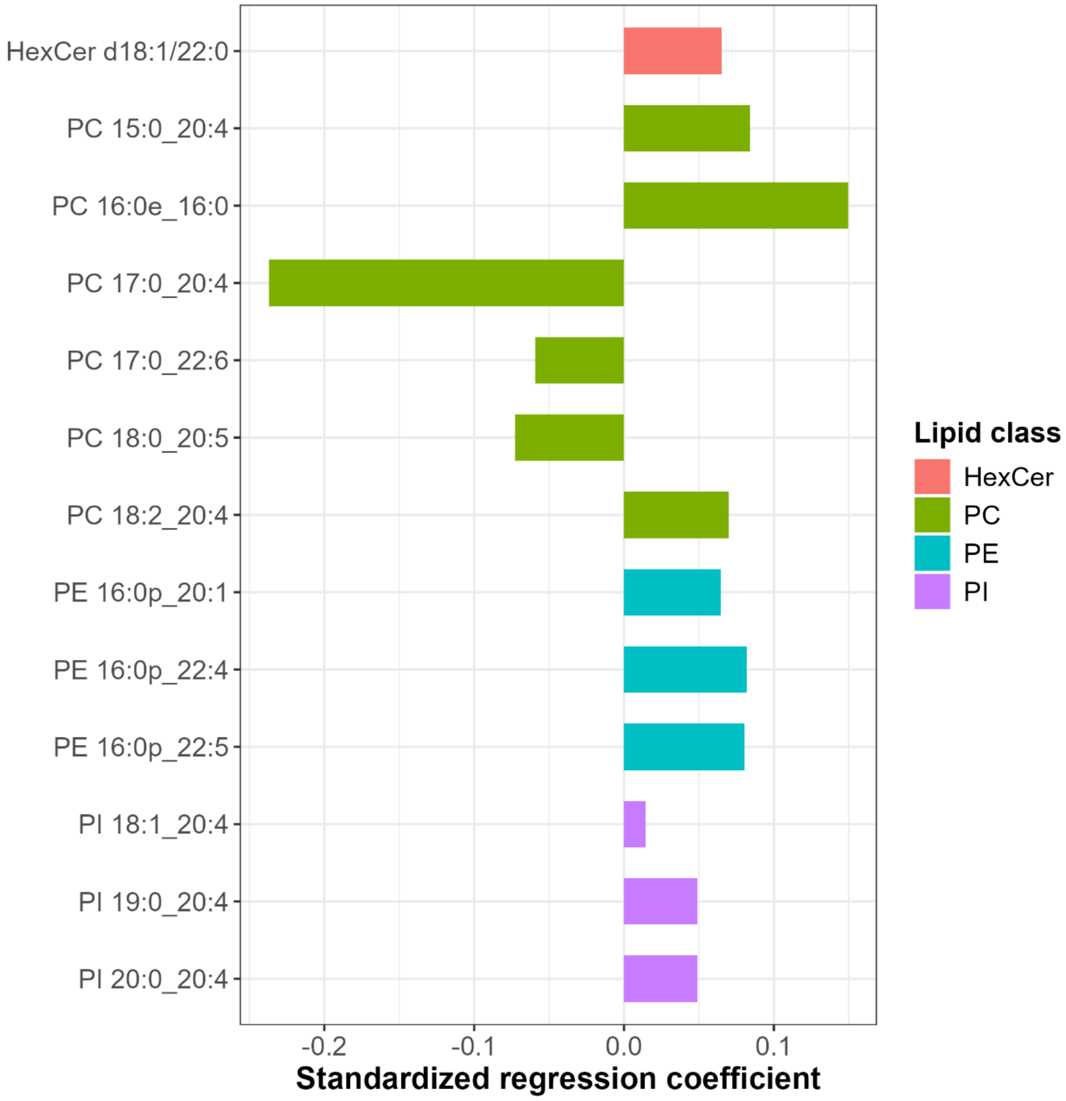

Figure S1. The results of the selection of the distinctive lipids related to flow-mediated vasodilation (FMD) using sparse-group LASSO regularized regression model.

This barplot shows the distinctive lipids selected using sparse-group LASSO regularized regression model and their standardized regression coefficient.

HexCer, hexosylceramide; PC, phosphatidylcholine; PE, phosphatidylethanolamine; PI, phosphatidylinositol; LASSO, least absolute shrinkage and selection operator.

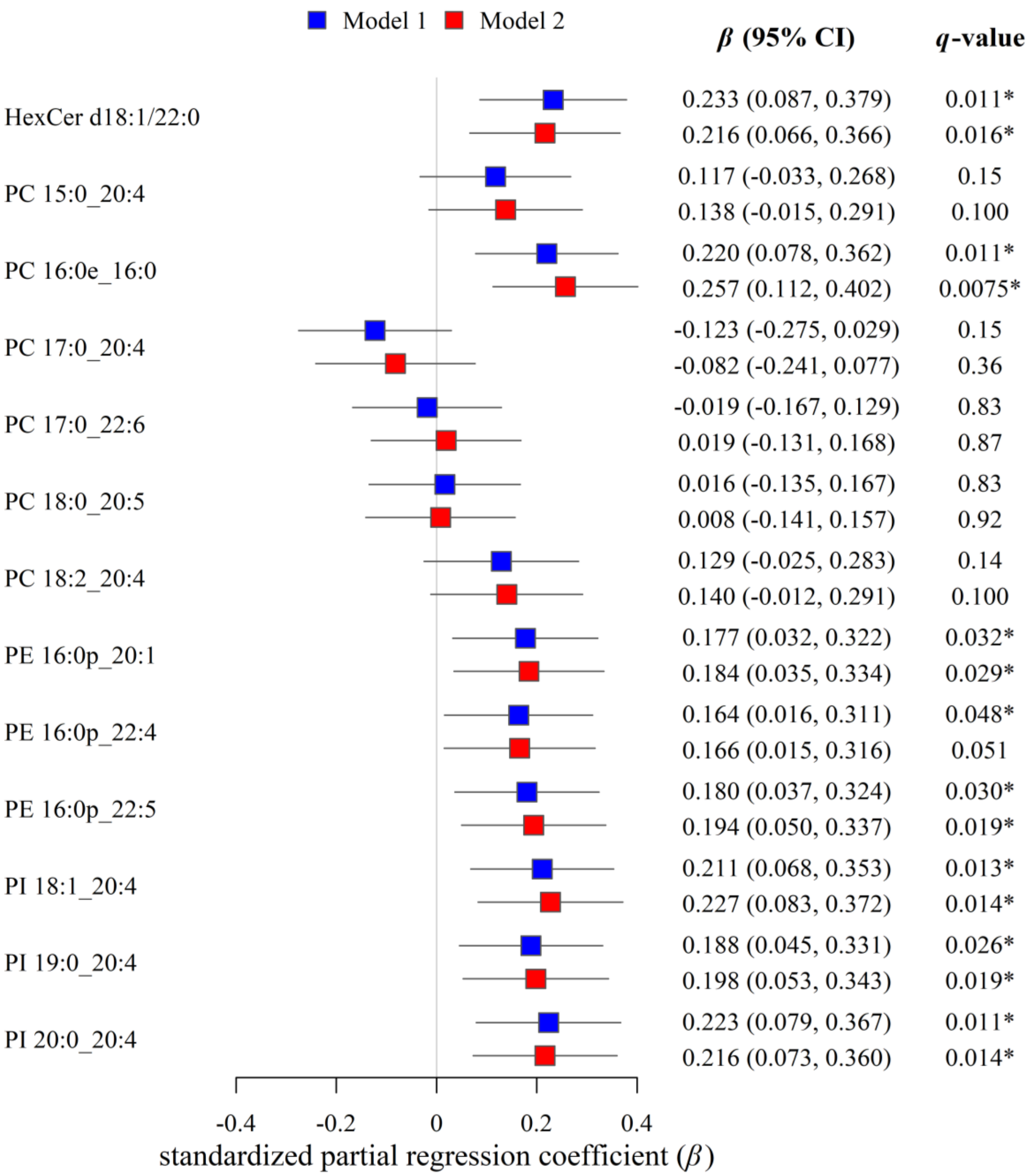

Figure S2. Associations between selected lipids and flow-mediated vasodilation (FMD) in patients without a history of coronary artery disease.

Linear regression analysis was performed to evaluate the association between the distinctive lipids selected using the sparse-group LASSO regularized regression model and FMD. Model 1 was adjusted for age and sex. Model 2 was adjusted for duration of diabetes, smoking status, BMI, hypertension, HbA1c level, statin use, and covariates in Model 1. The *q*-values (adjusted *p*-values) were calculated using the Benjamini-Hochberg method to account for multiple testing.

**q*-values indicating statistical significance (< 0.05).

β, standardized partial regression coefficient; 95% CI, 95% confidence interval; HexCer, hexosylceramide; PC, phosphatidylcholine; PE, phosphatidylethanolamine; PI, phosphatidylinositol; LASSO, least absolute shrinkage and selection operator.
